# A study of African swine fever virus in Regional VI of the Disease Investigation Center of Denpasar Bali in Indonesia

**DOI:** 10.14202/vetworld.2023.844-850

**Published:** 2023-04-21

**Authors:** Wayan Masa Tenaya, Ida Bagus Ngurah Swacita, Ketut Wirata, Made Damriyasa, Nengah Kerta Besung, Nyoman Suarsana, Tri Komala Sari, Kadek Karang Agustina

**Affiliations:** 1 Department of Disease Prevention, Veterinary Public Health, Faculty of Veterinary Medicine, Udayana University, Denpasar Bali of Indonesia, Jl. PB Sudirman, Denpasar, Bali 80234, Indonesia; 2 Disease Investigation Center, Regional VI Denpasar Bali, Jl. Raya Sesetan No. 266, Sesetan, Denpasar Selatan, Kota Denpasar, Bali 80223, Indonesia; 3 Laboratory of Clinical Pathology, Faculty of Veterinary Medicine, Udayana University, Denpasar Bali of Indonesia, Jl. PB Sudirman, Denpasar, Bali 80234, Indonesia; 4 Laboratory of Microbiology, Faculty of Veterinary Medicine, Udayana University, Denpasar Bali of Indonesia, Jl. PB Sudirman, Denpasar, Bali 80234, Indonesia; 5 Laboratory of Biochemical, Faculty of Veterinary Medicine, Udayana University, Denpasar Bali of Indonesia, Jl. PB Sudirman, Denpasar, Bali 80234, Indonesia; 6 Laboratory of Virology, Faculty of Veterinary Medicine, Udayana University, Denpasar Bali of Indonesia, Jl. PB Sudirman, Denpasar, Bali 80234, Indonesia

**Keywords:** African swine fever virus, epidemiology, macrophages, real-time polymerase chain reaction, vaccine

## Abstract

**Background and Aims:**

African swine fever (ASF) is a highly contagious viral disease that causes major economic losses due to morbidity and fatality rates of up to 100% in wild boar and domestic pigs. The disease emerged in Africa in 1921 and then entered several European countries by 1957. In Indonesia, the first outbreak of ASF in 2019 in North Sumatra killed thousands of pigs and quickly spread to 10 out of 34 pig-producing provinces, including Bali and Eastern Nusa Tenggara. As no commercial ASF vaccine is available, the disease has become endemic and continues killing pigs. This study aimed to investigate the epidemiological and virological studies of ASF virus (ASFV) conducted in 2020 and 2021 by the Disease Investigation Center Regional VI of Denpasar Bali, which covers three provinces in Indonesia, including Bali, Western Nusa Tenggara, and Eastern Nusa Tenggara.

**Materials and Methods:**

A total of 5402 blood samples were sent to the laboratory to detect ASFV infection using quantitative polymerase chain reaction (qPCR) and enzyme-linked immunosorbent assay tests. The virological studies were performed by culturing local ASFV isolates obtained from field cases in primary macrophages and confirmation of viral growth by qPCR.

**Results:**

The qPCR results show that only 156/4528 (3.4%) of samples originating from Bali and Eastern Nusa Tenggara were ASFV-positive with cycle threshold value of 18 to 23, while the virus was not detected in Western Nusa Tenggara. Of 874 serum samples tested, 114 (13%) were antibody positive and were all collected from the two ASFV-affected provinces in 2020. A Bali ASFV isolate (BL21) was isolated and characterized molecularly.

**Conclusion:**

These findings suggest that during the time of sampling, ASFV was detected only in Bali and East Nusa Tenggara but not in Western Nusa Tenggara. These findings support the symptomology of ASFV reported in the two regions. Moreover, BL21 may be useful for developing subculture-attenuated vaccines using commercial cell lines. However, the current study has some limitations namely the investigation was not performed during the initial outbreak and no pathological examination of internal organs was conducted.

## Introduction

African swine fever virus (ASFV), the causative agent of a highly contagious and lethal disease in wild boar and domestic pigs, is the most important virus within the family *Asfarviridae* [1, 2]. Within this family, ASFV is unique by virtue of being a nucleocytoplasmic large DNA virus with a multilayer structure and icosahedral morphology [3]. Its genome consists of 170–193 kb of double-stranded DNA that encodes 68 structural and more than 100 non-structural proteins [4]. The main cellular tropism of ASFV is the monocyte/macrophage lineages present in the bone marrow or in peripheral blood [5–7]. Although ASFV can infect a wide range of cell types originating from non-porcine species, these infections are generally non-productive, suggesting that the virus needs a specific receptor for effective internalization [8–10]. The virus enters macrophages through various mechanisms, such as dynamin-dependent and clathrin-mediated endocytosis, receptor-mediated endocytosis [11], micropinocytosis [10], phagocytosis [12], and the CD163 receptor [13].

Historically, ASF was first described in Kenya, Africa, in 1921, and then emerged in some European countries by 1957. At the beginning of 2020, the virus spread into 13 Asian countries [1, 14]. In Indonesia, an outbreak of ASF in 2019 in North Sumatra province killed about 42,000 pigs [15]. Subsequently, ASFV emerged in ten out of 34 pig-producing provinces, including Bali and East Nusa Tenggara provinces. The outbreak killed almost 3.5 million pigs [16]. Phylogenetic analysis showed that the ASFVs from North Sumatra, West Java, Vietnam, China, and Russia were identical, suggesting a common source of infection [17]. It is possible that the ASFVs found in Bali and East Nusa Tenggara are also genetically identical. The two of these provinces, as well as that of Western Nusa Tenggara, are supervised by the Disease Investigation Center region VI (DIC-Denpasar). ASFV has not been found in the Western Nusa Tenggara province. The outbreak of ASFV in Bali and East Nusa Tenggara was catastrophic, as it caused significant economic losses among pig farmers, especially in small-scale farms. Much of the damage was due to ASFV’s endemic phase and its uncontrolled spread to non-infected areas. Many ASFV-infected pigs survive and harbor the virus persistently in their blood and tissue for a long time. Such carrier pigs are a potential source of viral transfer [18]; moreover, carrier pigs can be studied to detect specific immune responses [19]. Data obtained from carrier pigs can be used to understand the epidemiology of ASFV better and to develop informed policies for eradication programs.

The only known measures for controlling ASF in the two infected regions were quarantining affected farms, farmer education, biosecurity, and strict sanitary procedures. However, the implementing these control strategies was ineffective; hence, the disease has continued. The rapid spread of the disease was mainly due to the lack of adequate vaccines. Efforts to develop ASF vaccines, such as gene-deleted vaccines, inactivated or subunit vaccines, and DNA-based vaccines, have thus far failed to protect pigs from the disease [20–24]. However, several live-attenuated vaccines have been shown to provide up to 100% protection [25, 26]. Pigs immunized with a live-attenuated ASFV (both naturally-attenuated and ASFV attenuated by passage through cell lines) can induce strong humoral and cellular responses with robust protection and can fully protect the immunized pigs against challenge from a lethal virus [25, 27]. Recent studies indicate that live-attenuated ASFV vaccines are the most advanced vaccine candidates ever made [2, 28]. Immunological studies have indicated that surviving pigs were resistant to challenges with certain ASFV isolates [5, 25, 29]. Moreover, serum or colostrum originating from convalescent pigs can reduce the severity of sickness, viremic titers, and mortality of acutely infected pigs after ASFV infection [25].

The above-mentioned studies strongly suggest that vaccine design is feasible, especially for live-attenuated vaccines. Therefore, this study aimed to determine the distribution of the disease, focusing on the three provinces; and to isolate and characterize an ASFV obtained locally from acute cases. The current results may be useful for developing subculture-attenuated vaccines using suitable cell lines [30].

## Materials and methods

### Ethical approval

This research has been approved by Udayana University ethical commission with letter No. B/217/UN14.2.9/PT.01.04/2022.

### Study period and location

The study was conducted from March 2020 to July 2021. The study area was carried out in three provinces namely Bali, Western Nusa Tenggara, and Eastern Nusa Tenggara with pig populations in 2020 were 398,291; 69,518 and 2,352,441, respectively.

### Epidemiological studies

Epidemiological studies using active and passive surveillance were performed shortly after the initial outbreak in 2019-2020 to determine the distribution of ASFV infection. A total of 5402 blood samples were analyzed in the DIC-Denpasar laboratory using the International Standard for laboratory testing methodology (ISO/SNI 17015:2018), coded as LP:123 IDN. Of the total samples, 4528 and 874 samples were analyzed by quantitative polymerase chain reaction (qPCR) and enzyme-linked immunosorbent assay (ELISA), respectively (Table-1). Fifty percentages of the samples were obtained directly from disease-affected farms by the surveillance team of DID-Denpasar (active surveillance). The other 50% of the samples were sent by district officials (passive surveillance). Detailed data were recorded by simple random sampling to determine the distribution of ASFV infection.

**Table-1:** Total and origin of samples used in this study.

Provinces	Year 2020		Year 2021	
	
	EDTA-blood	Serum	EDTA-blood	Serum
Bali	3080	451	376	-
Western Nusa Tenggara	-	-	70	-
Eastern Nusa Tenggara	782	423	220	-
Total	3862	874	666	0

The samples were tested using the laboratory standard protocol for real-time PCR (qPCR) and ELISA tests to detect specific ASFV-DNAs and humoral antibody response, respectively (https://bbvdps.ditjenpkh.pertanian.go.id/). qPCR=Quantitative polymerase chain reaction, ELISA=Enzyme-linked immunosorbent assay, ASFV=African swine fever virus

### Virological studies

#### Isolation and culture of macrophages

Swine macrophages were prepared from a 3-month-old uninfected piglet (determined by a negative PCR test) provided by DIC-Denpasar. All tissue culture work was performed in a type II biocontainment safety facility. Ten milliliters of whole blood were collected aseptically from the jugular vein using an EDTA tube. Macrophages were isolated from the blood using published methods [31–33] with some modifications. Briefly, 5 mL of the whole blood was mixed with 5 mL of balanced-salt medium (BSM, ThermoFisher Scientific, USA) in a 12 mL sterile plastic tube (Iwaki, Japan), which was carefully placed on top of a 50 mL conical tube containing 10 Ficoll-Paque PLUS (Cytiva, USA). The tube was centrifuged at 400× *g* for 30–40 min at 18°C–25°C. Subsequently, plasma was discharged, and the buffy coat containing peripheral blood mononuclear cells was carefully aspirated and mixed with three volumes of BSM in a 50 mL conical tube before being centrifuged at 350× *g* for 10 min at 18°C–25°C. The supernatant was discarded and the cell pellet was washed with BSM twice. Finally, the cell pellet was resuspended with 50 mL of complete Dulbecco’s modified eagle medium (CDMEM, ThermoFisher Scientific) containing 0.1 mL of heat-inactivated fetal calf serum, 1 μg of gentamicin, and 0.2 μg of amphotericin B per mL. The cell concentration was adjusted to 10^6^–10^7^ cells/mL by diluting with the CDMEM. The viability of the cells was >95% by keeping them at 5°C not more than 30 min before transferring them into a tissue culture flask

#### Culturing macrophages

The cell suspensions described above were dispensed into five sterile disposable 75-cm tissue culture flasks (Iwaki) and subsequently incubated in a CO_2_ incubator at a humid atmosphere at 37°C for 2 h. The humidity setting was done by placing a container with sterile water under the tray at the bottom of the incubator. The culture supernatant, which contained non-monocytes and non-adherent cells, was carefully aspirated and replaced with a new CDMEM. The flasks were then incubated for 7–14 days. Flasks were monitored daily for cell growth and any contamination. Every 3 days, the spent medium was replaced with a new CDMEM. Macrophages in the confluent phase were considered ready to be infected with ASFV isolates.

### Culturing ASFV isolates

African swine fever virus isolates were adapted using a previously published method [33], with slight modifications. Four ASFV isolates from four different districts in Bali were obtained from 4 infected pigs showing typical clinical signs of ASFV infection attesting positive on qPCR assays. The spleen of each ASFV-infected pig was aseptically removed, and the presence of infection in each spleen was reconfirmed by qPCR. The tissue was then made into a 10% suspension with cDMEM and passed through a 0.22 μm filter and used as an inoculant. Before inoculation, the culture supernatants from tissue culture flasks containing confluent macrophages were aspirated and washed twice with sterile PBS. Then, 5 mL of the inoculant was added to the four flasks. In a fifth flask, we added 5 mL of sterile PBS, and this flask served as a control. Flasks were incubated for 2 h for viral absorption; then, the non-absorbed viruses were washed away. Fresh CDMEM medium was added; then, the culture was incubated in a humid atmosphere at 37°C for 3 days. All flasks were monitored daily for the presence of cytopathic effects (CPEs) in the infected cultures.

### Isolation of ASFV-DNAs and qPCR analysis

DNAs for qPCR analysis were purified from the supernatants of the ASFV-infected tissue cultures that showed CPEs and from the uninfected control. DNA was extracted using a DNeasy^®^ blood and tissue kit isolation kit (Qiagen, UK), according to the manufacturer’s instructions, with minor modifications. The extracted DNA was kept at −20°C until subsequent TaqMan PCR (ThermoFisher Scientific) using the following forward (5’CTGCTCATGGTATCAA TCTTATCGA’3) and reverse (5’ GATACCACA AGATCRGCCGT’3) primers and probe (6FAM CCACGGGAGGAATACCAACCCAGT TAMRA). This PCR targets the conserved regions at the 3’-end of VP72 gen [34]. Twenty-five microliters of master mix reaction contained; 5.0 µL of PCR%-free water; 12 µL of TaqMan universal, Primer Probe (5 mg); 1 mL, primer forward (18 mg); 1 mL, primer reverse (18 mg); and 5 mL of DNA template. The DNAs were amplified for 45 cycles, with each cycle consisting of denaturation at 50°C for 2 min, amplification at 95°C for one min, and extension at 95°C for 15 s. Finally, the plate was held at 5°C until the fluorescence signal was measured using an ABI Prism 7200 sequence detection system (Applied Biosystems, USA).

## Results

### Epidemiological studies

A total of 5332 samples (98.7%) were from Bali and Eastern Nusa Tenggara provinces, and only 70 EDTA-blood samples (1.3%) were from Western Nusa Tenggara, which is a region with the lowest pig population. No ASFV infection has been observed in Western Nusa Tenggara based on risk-based surveillance. Of the total DNA samples tested, only 156 (3.4%) were qPCR-positive for ASFV; all positive cases were detected in Bali and Eastern Nusa Tenggara provinces. The proportion of DNA-positive samples obtained from Eastern Nusa Tenggara was higher (13%) than those from Bali (0.8%). Within these two provinces, 114 out of the 874 (13%) serum samples were ELISA-positive (Table-2).

**Table-2:** Total of qPCR- and ELISA-positive samples detected in this study.

Provinces	Year 2020		Year 2021	
	
	EDTA-blood	Serum	EDTA-blood	Serum
Bali	16/3,080	29/451	11/376	-
Western Nusa Tenggara	-	-	0/70	-
Eastern Nusa Tenggara	86/782	85/423	43/220	-
Total	102/3862 (2.17%).	114/874 (13%)	54/666 (8.1%)	0

qPCR=Quantitative polymerase chain reaction, ELISA=Enzyme-linked immunosorbent assay

### Tissue culture adaptation of monocyte/macrophages

**Figure-1:**
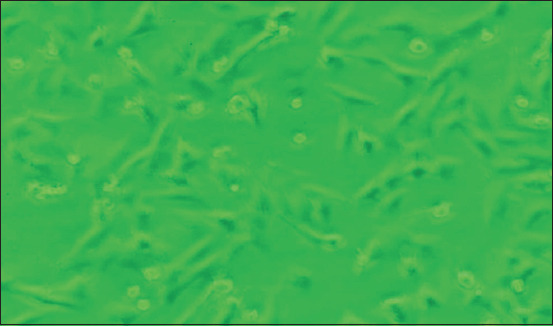
Macrophages on 5 days of culture stages with a typical non-round form of macrophages attached strongly on the bottom of the flask (100×).

During preliminary studies, we compared two powder-based media, complete Roswell Park Memorial Institute (RPMI), and Dulbecco’s modified eagle medium (DMEM), to determine the best conditions for culturing the ASFV-target cells. These studies found that RPMI medium supports less cell growth (data not shown). Similarly, we investigated two techniques for the isolation of monocyte/macrophages, that is, isolation from ascites fluids of an anesthetized-normal piglet and isolation from peripheral blood. We observed problems with the use of ascites fluids due to mycoplasma contaminations; therefore, we did not use this technique in subsequent research. The DMEM (ThermoFisher Scientific) strongly supported monocyte/macrophage growth prepared from peripheral blood. This procedure was considered efficient and simple; thus, we used it throughout this work. Typical macrophage-like cells appeared as non-round, and they proliferated on a mixed monolayer cell sheet consisting of islands of polygonal cells that were strongly attached to the bottom of the flask. These cells were clearly observed after 5 days of culturing (Figure-1). After approximately 2–3 weeks of growth, confluent cells were observed, and we considered these cells as ready for infection by the ASFV isolates.

### Culturing ASFV isolates and confirmation with qPCR

**Figure-2:**
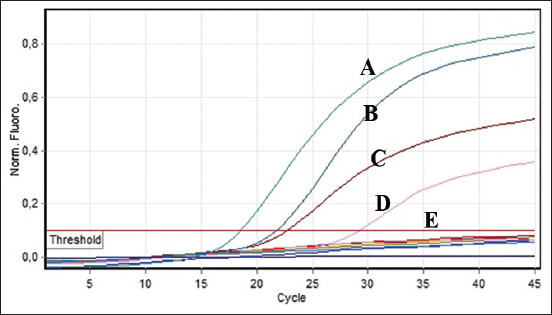
Quantitative polymerase chain reaction analsis of ASFV isolated from ASFV-infected tissue culture and DNA samples originated from ASF-suspected field cases in Bali. A and B were DNAs from field cases. (C) DNA-positive control. (D) DNA of the ASFV-infected tissue culture. (E) Negative control (below a cycle threshold value). ASFV: African swine fever virus, ASF: African swine fever.

We observed CPEs 3 days after inoculating prepared ASFV isolates onto confluent macrophages. The infected macrophages were destroyed, with most of the cells floating on the surface of the medium. We detected no bacterial or mycoplasmal contamination. We confirmed ASFV infection by performing a qPCR assay on the supernatant of the culture medium. The qPCR-positive results suggested the presence of a specific ASFV isolate, which we named as BL21. This isolate was then characterized, and revealed that qPCR results of the culture supernatant and that of the field cases were identical. As expected, a negative control yielded negative qPCR results (Figure-2).

## Discussion

By December 2021, 10/34 provinces of Indonesia were infected by ASFV. Two of these provinces, that is, Bali, and East Nusa Tenggara, were under the supervision of DIC-Denpasar. Meanwhile, ASFV was not detected in Western Nusa Tenggara [35]. Epidemiological surveillance was conducted shortly after the outbreak, which was accomplished by collecting many samples from ASF-contaminated locations. One aim of the surveillance was to determine the prevalence of ASFV infection, which constitutes information that may be useful for designing evidence-based control strategies. This was the first surveillance that was conducted to detect clinically healthy carriers that were a likely reservoir of ASFV in nature that can potentially hamper eradication programs [18]. As shown in Table-2, only 3.4% of the total of 4528 DNA samples were positive for ASFV DNA, based on qPCR. Although the proportion of ASFV-positive pigs was quite small, this nevertheless constitutes a serious risk, because carrier animals can potentially contribute to the persistence of ASFV infection [18]. A huge number of DNA samples (4,528) were collected from infected provinces and the surveillance was performed shortly after the first outbreak. It is possible that the majority of ASFV-infected pigs had died due to the high morbidity and mortality rates of ASFV infection during the initial outbreak; and only a minority of them, acting as carrier animals, survived. Moreover, the detection of ASFV-DNAs from blood is considered less sensitive than detection from internal organs. For example, a study to detect ASFV in the tissues of asymptomatic pigs conducted in Africa [36] showed that 15.9% of 44 internal organs obtained from clinically normal pigs were positive for ASFV based on PCR assays; moreover, one pig with negative ELISA results tested was also positive for ASFV based on PCR conducted postmortem. These results suggest that ASFV may be hidden in the internal organs of pigs [36]. In this study, only 114 out of the 874 (13%) serum samples were positive for ASFV based on ELISA. Ideally, all ELISA-positive should also test positive with qPCR; however, we did not perform such confirmatory testing. In future studies, samples for qPCR and ELISA should be collected from the same pigs and tested in parallel. Results of our risk-based surveillance show that a majority (98.7%) of the samples were collected from Bali and Eastern Nusa Tenggara provinces (ASFV-infected provinces), while about 1.3% of the serum samples were obtained from Western Nusa Tenggara, which had the lowest pig population that yielded no positive ASFV infection results (Table-2). This data may support ASFV control measures by providing useful information for banning the transport of pigs from infected to naïve districts. Future surveillance work should validate diagnostic procedure by detecting ASFV DNA obtained from the internal organs of ASFV-suspected pigs. During large-scale testing, serological assays can be used for screening samples and then qPCR can be used to confirm ASFV-positive pigs, especially before transport to other districts [37].

The rapid outbreak and continuous spread of ASFV infection have revealed a challenge for effective disease control. Aside from biosecurity measures to reduce the risk of viral deployment, one of the top priorities for disease control is the availability of effective vaccines [24]. To propagate ASFV, we initially prepared swine primary macrophages, in which we were able to adapt well and grow robustly. Early propagation of locally derived ASFV isolates in the confluent cells was successful on 3 days post-infection, with the infected cells showing CPEs. Almost all cells were damaged and floated to the surface, in contrast to cells in the control flask. We detected no microbial contamination (data not shown). Using qPCR, we confirmed that the supernatant of the cultures was positive for ASFV. In this study, we successfully isolated and characterized an isolate named BL21, which has been stored in liquid nitrogen for future study. This is the first report to confirm the presence of adapted ASFV in a tissue culture system. Confirmation was based on qPCR in parallel with ASFV isolated from the spleen of naturally infected pigs, although the cycle threshold value was <20 (Figure-2). Confirmation of ASVF through qPCR was more specific and sensitive than ELISA test; this method can confirm DNA of the virus, although others have demonstrated the presence of ASFV proteins using an immunohistochemical test [38]. In the previous work, porcine monocytes/macrophages were the only *in vitro* system that successfully propagated ASFV, although wild-type ASFV obtained from field cases could not be propagated directly in commercial cell lines [33]. Based on this result we assumed that BL21 (which we successfully cultured in macrophages) can be adapted to commercial cell lines. If successful, then this system can provide an adequate vaccine candidate that is safer than live-attenuated ASVF vaccine and thus protect pigs against ASFV.

## Conclusion

We report here that the current prevalence of ASFV infection in the regional VI of Disease Investigation Center Denpasar Bali of Indonesia is 3.4%. ASFV infection was detected only in Bali and Eastern Nusa Tenggara provinces. Moreover, we were able to propagate a locally derived ASFV isolate, which we designated BL21. This isolate has been successfully characterized, and it may be useful for the development of subculture-attenuated vaccines using commercial cell lines. The main limitation of this study is that sample collection was performed only after the outbreak period. In the future, samples should be collected during the acute phase of the disease outbreak, to obtain a more accurate prevalence estimate.

## Authors’ Contributions

WMT, IBNS, and KKA: Designed and conducted the research, analyzed data, and drafted the manuscript. KW, MD, NKB, NS, and TKS: Performed qPCR, analyzed the data and drafted and revised the manuscript. All authors have read, reviewed, and approved the final manuscript.
